# Morphometric profile of the localised renal tumors managed either by open or robot-assisted nephron-sparing surgery: the impact of scoring systems on the decision making process

**DOI:** 10.1186/1471-2490-13-63

**Published:** 2013-11-27

**Authors:** Tarık Esen, Ömer Acar, Ahmet Musaoğlu, Metin Vural

**Affiliations:** 1 School of Medicine, Koc University, Istanbul, Turkey; 2 Department of Urology, VKF American Hospital, Istanbul, Turkey; 3 Department of Radiology, VKF American Hospital, Istanbul, Turkey

**Keywords:** Renal tumor, Open surgery, Robotics, Morphometry

## Abstract

**Background:**

Nephrometric scoring systems aim to improve the manner in which tumoral complexity is measured and reported. Each system provides a way to objectively measure specific tumor features that influence technical feasibility. In this study we aimed to determine how nephrometric scoring systems tailored our approach to the surgical treatment of localised renal masses.

**Methods:**

Charts of the patients with localised renal tumors, who were managed by either open or robot-assisted nephron-sparing surgery between May 2010 and June 2012, were retrospectively reviewed. Nephrometric scores [radius, exophytic/endophytic, nearness, anterior/posterior, location (R.E.N.A.L.) score, preoperative aspects and dimensions used for anatomic (P.A.D.U.A.) classification and centrality index (C-index)] were calculated based on preoperative imaging findings. Perioperative data were recorded. Morphometric characteristics of the renal masses were compared. Additionally, the difference between surgical alternative subgroups in terms of morphometric variables and the predictive power of each scoring system in determining the details of the surgical plan were investigated. Furthermore, surgical preferences in different nephrometric categories were compared.

**Results:**

Mean R.E.N.A.L. and P.A.D.U.A. scores of the tumors treated with robotic surgery were significantly lower than those managed by open surgery. R.E.N.A.L. nephrometry score showed significant differences between most of the surgical alternative subgroups. P.A.D.U.A. and C-index differences were significant only between robotic off-clamp and open clamped cases. Tumors that required open conversion had significantly higher mean R.E.N.A.L. and P.A.D.U.A. score. High R.E.N.A.L. score (cut-off: 6.5) and high P.A.D.U.A. score (cut-off: 7.5) were found to be significant predictors of the surgical route. Significantly more tumors with moderate R.E.N.A.L. score were managed through the open approach, while the significant majority of those with low R.E.N.A.L. and low P.A.D.U.A. score were operated by robotic assistance.

**Conclusions:**

R.E.N.A.L. and P.A.D.U.A. scores influenced our surgical treatment strategy for localized renal masses. High R.E.N.A.L. and P.A.D.U.A. scores increased the likelihood of an open NSS.

## Background

With recent advances in imaging modalities, the incidental detection of localised renal cell carcinoma (RCC) has increased by almost 3.7% per year over the past several decades [1]. Over 60% of renal masses detected each year are <4 cm in size [2]. Concurrently, the incidence of systemic conditions favoring the development of chronic kidney disease, such as hypertension and diabetes mellitus, are on the rise [3,4]. Considering the facts that, partial nephrectomy has similar oncologic outcomes to that of radical surgery for T1 tumors and renal insufficiency is associated with adverse cardiovascular outcomes, current evidence suggests that localised renal cancers are best managed by nephron-sparing surgery rather than by radical nephrectomy, whenever technically feasible [5].

Tumoral complexity remains the primary parameter according to which urologists determine the surgical approach and treatment strategy regarding renal masses. Technical details such as the route (open vs. minimally invasive) and the decision to cease renal blood flow temporarily during mass excision (warm ischemia vs. no ischemia) are under the influence of tumor characteristics. Morphometric scoring systems (R.E.N.A.L., P.A.D.U.A. and C-index) have been developed in an effort to standardize the nomenclature while discussing how “challenging” the tumor is [6-8]. In this study, we aimed to determine how nephrometric scoring systems, tailored our approach in nephron-sparing surgery (NSS).

## Methods

Robotic technology has been installed at our hospital in May 2010 after which we have performed both open and robot-assisted NSSs. After IRB approval (IRB protocol no: 2013.189.IRB2.58), we reviewed the charts of the patients who have undergone NSS (open and robot-assisted) between May 2010 and June 2012 in our clinic. Data were retrospectively collected from a prospectively structured database. All operations were carried out by a single surgeon (TE), who is highly experienced in open NSS but has never performed pure laparoscopic surgery. He has accomplished a direct transition from open to robot-assisted laparoscopic surgery. Patient characteristics were analyzed, including demographic data, past medical history, mode of presentation, comorbidities and American Society of Anesthesiologists (ASA) score.

Radiologic characteristics of the renal masses were scored retrospectively by a senior radiologist and urologist according to C-index method [6], R.E.N.A.L. nephrometry system [7] and P.A.D.U.A. classification [8], as described elsewhere. The radiologist and the urologist were blinded with regards to surgical approach and technical details while they were calculating the morphometric scores. Based on preoperative radiologic findings, none of the patients had lymph node involvement or distant metastasis.

A total of 32 and 23 patients underwent robot-assisted and open NSS, respectively after the introduction of robotic technology in our clinic. Coexisting systemic medical problems (Hypertension, diabetes mellitus, etc.) were present in 38% (n = 21/55) of the patients, while 1 patient in the robotic group and 3 patients in the open surgery group had a solitary kidney.

Open nephron-sparing surgery (ONSS) was performed using the intercostal (between 11^th^ and 12^th^ ribs) extraperitoneal flank approach, as previously described [9]. Briefly, after adequate exposure of the kidney, Gerota’s fascia was opened and perinephritic fatty tissue was dissected off the renal surface. Ureter and the vascular pedicle were marked with vessel loops. The decision about hilar clamping was given perioperatively according to in-situ findings and preoperative radiologic data. We did not implement cold-ischemia in any of these open NSS’s. Tumors were removed via enucleoresection [10]. Bleeding from the tumor bed was controlled with 3/0 polyglactin interrupted sutures and parenchyma was adapted with 2/0 monofilament running sutures, over a surgical bolster.

All robot-assisted nephron-sparing surgeries (RANSS) were performed using the da Vinci surgical system (Intuitive Surgical, Inc., Sunnyvale, CA) with a 5-port approach, including two 8 mm ports for robotic instruments, one 12 mm port for the robotic scope, and 2 ports for the bedside assistant. RANSS’s were carried out through the transperitoneal route with the patient in flank position. After colonic mobilization, Gerota’s fascia was opened and tumor was adequately exposed. The decision to clamp renal pedicle was given during the operation, based on CT and/or MR images and intraoperative findings. If there was such a need, the renal artery was occluded with an external vessel loop secured with a hem-o-lok clip over a silicone tube [11]. After demarcating tumor margins with electrocautery, resection was carried out using cold-scissors. All tumors were enucleoresected leaving a minimal margin of normal parenchyma. Tumor bed was oversewn with 3/0 polyglactin sutures (in case of pelvicalyceal violation) and parenchyma was approximated using the “sliding clip” technique.

Operative data consisted of total operative time, estimated blood loss (EBL), warm-ischemia time (WIT) and adverse events. Final pathology reports were analyzed and thirty-day Clavien grade 2 and higher complications were recorded [12].

We compared the morphometric characteristics of the renal masses managed by open and robot-assisted NSS. In addition, we investigated if nephrometric scores differed significantly between surgical alternative subgroups (robotic clamped, robotic off-clamp, open clamped, open off-clamp). Furthermore, surgical preferences in different nephrometric categories were compared. We also tested the predictive power of each scoring system in determining the details (route, hilar clamping) of the surgical plan.

Statistical calculations were performed with the *t* test, Fisher’s exact test and univariate linear models which were provided by the commercially available software (SPSS version 20).

## Results

Demographic data of the patients enrolled to the study is shown in Table 1. Patients in the open surgery group were significantly older (p = 0.049). However, the difference between mean ASA scores was insignificant. Majority (72% in the robotic surgery group vs. 82.6% in the open surgery group, p = 0.52) of the tumors were discovered incidentally on imaging studies that were ordered for non-urologic complaints.

**Table 1:** Differences between open and robotic groups in terms of demographic data, tumor characteristics and perioperative outcomes

		**RANSS**	**ONSS**	**P value**
Mean age (years)		51.5 ± 13.4 (range = 30–76)	58.5 ± 10.25 (range = 31–82)	**0.049**
Gender (female/male)		9/23	8/15	N.A.
Mean A.S.A. score		1.47 ± 0.51 (range = 1–2)	1.43 ± 0.59 (range = 1–3)	0.819
Mean tumor size (cm)		4.25 ± 3.0 (range = 1.2-15)	4.27 ± 2.27 (range = 0.5-9.5)	0.976
Tumor morphometry (mean values)	R.E.N.A.L.	6.15 ± 2.04 (range = 4–10)	8 ± 1.5 (range = 5–10)	**0.0006**
	P.A.D.U.A.	7.53 ± 1.81 (range = 6–11)	8.56 ± 1.44 (range = 6–11)	**0.027**
	C-index	1.49 ± 0.43 (range = 0.7-2)	1.25 ± 0.5 (range = 0–2)	0.066
Mean operative duration (min)		154.3 ± 55.3 (range = 70–270)	126.09 ± 33.51 (range = 75–220)	**0.034**
Mean EBL (ml)		189.1 ± 165.4 (range = 100–1000)	239.13 ± 102.2 (range = 100–500)	0.204
Transfusion (n)		5 (15.62%)	1 (4.34%)	0.383
Hilar clamping (n)		8 (25%)	6 (26.08%)	1.0
Mean WIT (min)		20.62 ± 2.19 (range = 16–24)	16.50 ± 5.21 (range = 12–23)	0.064
Mean duration of hospitalization (days)		4.06 ± 1.29 (range = 3–7)	4.6 ± 1.75 (range = 3–10)	0.188
30-day Clavien grade ≥ 2 complications (n)		6 (18.75%)	4 (17.39%)	0.988

Tumor size ranged between 0.5 – 15 cm and the mean diameter did not differ significantly between two groups. Mean R.E.N.A.L. and P.A.D.U.A. scores were significantly higher in the open surgery group (Table 1).

Operative findings are summarized in Table 1. Robot-assisted operations lasted significantly longer than their open counterparts. Although mean EBL amount was higher in the open surgery group, transfusion rate was higher in the robot-assisted surgery group. However, these differences were not statistically significant. Renal hilum was clamped in 6 (26%) and 8 (25%) patients in the open and robotic groups, respectively. The difference between mean warm-ischemia times was insignificant.

Lengths of hospital stay and complication rates were similar between groups. Blood transfusion (n = 3), double-j catheter insertion because of urinary extravasation (n = 1) and nasogastric tube insertion due to ileus (n = 2) were the recorded Clavien grade ≥ 2 complications in the robotic group. One patient in the ONSS group, who had a 9 cm solid mass bearing solitary kidney, suffered from a total of 3 Clavien grade ≥ 2 complications (blood transfusion, double-j insertion, temporary hemodialysis) postoperatively. Blood transfusion in another patient was the remaining Clavien grade ≥ 2 complication in the open surgery group.

Open conversion (15.6%, n = 5) was necessary because of tumor size in 2, difficulty in dissection in 1 and uncontrollable bleeding in 2 cases. Converted cases had significantly higher mean R.E.N.A.L. (9.0 ± 0.71 vs. 5.63 ± 1.76, p = 0.0002) and P.A.D.U.A. scores (9.6 ± 0.89 vs. 7.15 ± 1.68, p = 0.003) than the cases, which were accomplished with robotic assistance. However, the difference was insignificant in terms of mean C-index value (1.42 ± 0.42 vs. 1.50 ± 0.44, p = 0.698).

Histopathologic diagnosis was renal cell carcinoma (RCC) in 42 patients, (76%) with clear cell variant being the most common (n = 32, 76%) subtype. Angiomyolipoma (n = 5) represented the most frequently diagnosed benign lesion. Surgical margins were free of tumoral infiltration in all patients.

**Figure 1:**
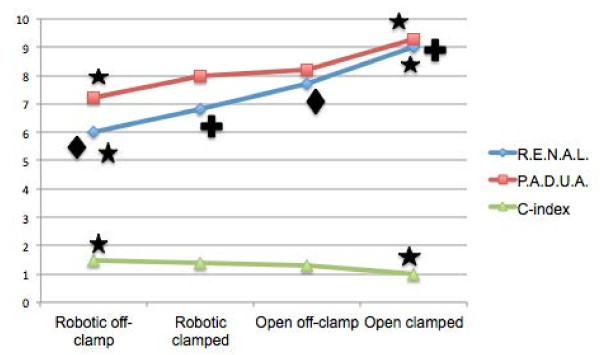
**Morphometric data of the renal tumors managed with open or robotic approach under ischemic and non-ischemic conditions.** ★: Significant differences between robotic off-clamp vs. open clamped cases in terms of R.E.N.A.L. (5.96 ± 1.97 vs. 9.0 ± 0.63, p = 0.0009), P.A.D.U.A. (7.38 ± 1.76 vs. 9.5 ± 0.84, p = 0.008) and C-index (1.52 ± 0.40 vs. 1 ± 0.63, p = 0.017) values. ♦: Significant difference between robotic off-clamp vs. open off-clamp cases in terms of R.E.N.A.L. score (5.96 ± 1.97 vs. 7.65 ± 1.58, p = 0.005). **+**: Significant difference between robotic clamped vs. open clamped cases in terms of R.E.N.A.L. score (6.75 ± 2.31 vs. 9.0 ± 0.63, p = 0.04).

The relationship between surgical alternatives and nephrometric data is schematized in Figure 1. Tumors managed with open NSS under warm ischemia, had the highest mean R.E.N.A.L. and P.A.D.U.A. score and the lowest mean C-index value (p < 0.05). Surgical alternative subgroups showed significant differences between each other with regard to R.E.N.A.L. nephrometry score. P.A.D.U.A. and C-index differences were significant only between the tumors dealt with non-ischemic robot-assisted surgery and open surgery under ischemic conditions (Figure 1).

After investigating the management strategy of the tumors in each R.E.N.A.L. and P.A.D.U.A. category (low, moderate, high), it was found out that, significantly more patients with low R.E.N.A.L. score and low P.A.D.U.A. score were managed with robot-assisted surgery whereas the majority of those with moderate R.E.N.A.L. score were treated with open NSS (Table 2).

**Table 2:** Number of patients in each R.E.N.A.L. and P.A.D.U.A. category operated by either open or robot-assisted surgery

		**ONSS (n = 23)**	**RANSS (n = 32)**	**P value**
R.E.N.A.L. score (n)	Low	4	20	**0.001**
	Moderate	16	10	**0.006**
	High	3	2	0.639
P.A.D.U.A. score (n)	Low	4	19	**0.002**
	Moderate	11	7	0.07
	High	8	6	0.2

R.E.N.A.L. system; low score: 4–6, moderate score: 7–9, high score: 10–12.

P.A.D.U.A. system; low score: 6–7, moderate score: 8–9, high score: ≥10.

**Figure 2:**
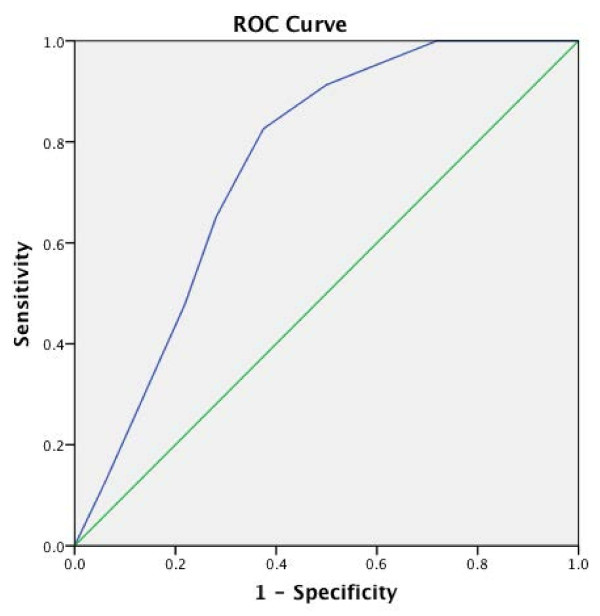
Receiver operating characteristics of R.E.N.A.L. score with regard to surgical route prediction (Cut-off: 6.5, AUC: 0.755, p = 0.001, sensitivity: 82.6, specificity: 62.5).

**Figure 3:**
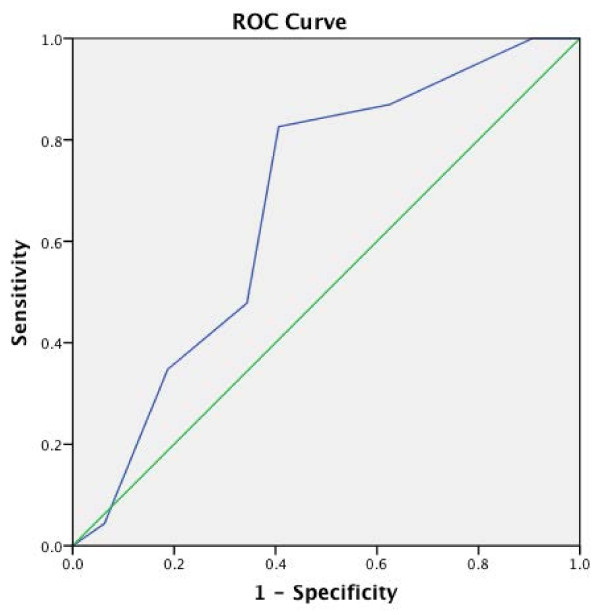
Receiver operating characteristics of P.A.D.U.A. score with regard to surgical route prediction (Cut-off: 7.5, AUC: 0.673, p = 0.028, sensitivity: 82.6, specificity: 59.4).

On univariate analyses, R.E.N.A.L. and P.A.D.U.A. scores were found to be significant predictors of the operative route (open vs. robot-assisted). R.E.N.A.L. score (cut-off value: 6.5) predicted the likelihood of open route with a sensitivity of 82.6% and specificity of 62.5% (Figure 2). P.A.D.U.A. score (cut-off value: 7.5) predicted the same outcome with a sensitivity of 82.6% and specificity of 59.4% (Figure 3). None of the morphometric systems predicted the status of renal perfusion during enucleoresection (clamped vs. off-clamp) with adequate statistical significance.

## Discussion

Nephron-sparing surgery has been accepted as the ideal treatment of localised RCC, given the similar oncological outcomes to that of radical surgery [13-17]. However, imaging studies may sometimes question the feasibility of NSS mostly because of either the size or the localization of the tumor. Moreover, without a structured and reproducible system for describing the relevant renal mass anatomy, treatment decisions will vary, depending on urologist’s training, biases, comfort levels, and individual experience.

Recently, three different scoring systems were developed to serve as a common vocabulary when discussing anatomic geometry and complexity of renal masses. Preoperative aspects and dimensions used for anatomic (P.A.D.U.A.) classification and R.E.N.A.L. (radius, exophytic/endophytic, nearness, anterior/posterior, location) scoring systems involve similar components and methodology, enabling a comprehensive description of the tumor size, polarity, anterior/posterior location and proximity to the collecting system [7,8]. The centrality index (C-index), however, is a completely different system that involves a relatively complex mathematical concept and characterizes tumor centrality based on the ratio of the distance between the tumor and kidney center and tumor radius [6]. Currently, there is no clear consensus favoring the utility of any score or index over the others.

Nephrometric scoring systems have also been associated with certain measures of operative complexity such as ischemia time and complication rates. Waldert et al. reported a significant association between high P.A.D.U.A. scores (≥ 10) and increased WIT (22 vs. 34 minutes) [18]. Similarly, Simmons et al. stated that tumors with a C-index of ≤ 1 had a 2.3-fold risk of prolonged WIT (≥ 35 minutes) compared with tumors with a C-index ≥ 1 [6]. Each unit increase in R.E.N.A.L. score was associated with a 35% increased risk of postoperative urine leakage in a study conducted by Bruner et al. [19]. Another study highlighted the significant increase in complication rates with increasing P.A.D.U.A. score among patients undergoing partial nephrectomy. Tumors with scores of 8 to 9 had a hazard ratio of 14.5 for postoperative complications compared with tumors with scores of 6 to 7. Tumors scoring ≥ 10 had a hazard ratio of 30.6 [8].

Apart from the documented power in predicting perioperative outcomes, these scoring systems also have the potential to influence surgical preferences, which covered the scope of this article. According to our results, mean R.E.N.A.L. and P.A.D.U.A. scores of the tumors managed by robotic assistance were significantly lower than those treated by open surgery. Although being lower in tumors managed by open approach, C-index values did not exhibit a statistically significant difference between open and robotic groups (Table 1). These differences may be due to selection biases, with more peripheral, exophytic, and smaller tumors being scheduled for robot-assisted surgery. However, these are the initial robot-assisted nephron sparing surgeries performed in our clinic by a surgeon who is experienced in open surgery.

Tumor size, estimated blood loss amounts and transfusion rates, which would be considered among the factors indirectly reflecting how “tough” the tumor was, did not differ significantly between open and robotic groups. Robot-assisted nephron-sparing surgeries lasted significantly longer than their open counterparts. However, this difference may not be regarded as a sign of tumoral complexity since recorded time in robotic surgeries included the “docking” maneuvers.

In our practice, the decision to occlude renal pedicle is given during the operation. Therefore, it might be regarded as an “in-vivo” validation of the preoperative morphometric information. Moreover, interrupting renal perfusion temporarily constitutes a major clinical concern since longer warm ischemia time has been associated with acute renal failure, decreased glomerular filtration rate and de-novo chronic kidney disease [20]. In order to clarify this issue we investigated the differences between surgical choice subgroups in terms of morphometric scores. We found a statistically significant difference between robotic off-clamp cases and open clamped cases with regard to each morphometric score (R.E.N.A.L., P.A.D.U.A. and C-index, Figure 1). However, only R.E.N.A.L. score demonstrated significant differences across most of the studied surgical alternative subgroups (robotic off-clamp vs. open off-clamp and robotic clamped vs. open clamped) (Figure 1).

Open conversion during minimally invasive surgery might be regarded as another way of expressing the challenging nature of the tumor being handled. In our series, those tumors, for which open conversion was inevitable, had significantly higher mean R.E.N.A.L. and P.A.D.U.A. scores than those who were successfully treated in a minimally invasive fashion.

On univariate analyses, P.A.D.U.A. and R.E.N.A.L. scores demonstrated sufficient predictive power in determining the route by which tumor was enucleoresected. High R.E.N.A.L. score (cut-off value: 6.5) and high P.A.D.U.A. score (cut-off value: 7.5) predicted the likelihood of an open NSS, with adequate statistical power. This finding was complemented by the significant difference between the number of patients in low, moderate R.E.N.A.L. and low P.A.D.U.A. categories with regard to the route that has been preferred for NSS (Table 2). In a similar study including more than 400 patients undergoing NSS, Canter et al. reported that patients undergoing open surgery had a significantly higher mean R.E.N.A.L. score, than those managed with minimally-invasive NSS (8.19 vs. 6.62, p < 0.0001) [21]. Recently, Rosevear et al. confirmed the predictive power of R.E.N.A.L score in terms of operative approach selection, with significantly more patients with low and high scores undergoing partial and radical nephrectomy, respectively in their cohort of 249 patients [22].

However, our findings do not mean that we preferentially use the R.E.N.A.L. or the P.A.D.U.A. system for preoperative morphometric assessment. Based on the available literature, there is no clear advantage of one scoring system over the others regarding surgical preferences and perioperative outcome. As the number of patients in each sub-category increase, the C-index differences that were stated as insignificant might gain statistical significance. Therefore, it is hard to draw strict conclusions about the superiority of R.E.N.A.L. or P.A.D.U.A. scoring systems based on our results.

Our study is unique in that it focuses on the utility of morphometric scoring systems in tailoring the surgical approach rather than perioperative outcomes or oncologic results. Moreover, only nephron-sparing surgeries performed by a single surgeon through the open or robot-assisted laparoscopic route were taken into consideration. At last but not the least, we tested the discriminative power of all three scoring systems that are currently being utilized across the globe. However, this data reflects the experience of a single surgeon, who is proficient in open NSS and currently in the initial phase of the learning curve for robotic NSS, which may limit the reproducibility of our findings. Our statistical findings are also handicapped by the retrospective study design and small sample size. Another criticism is the lack of strictly defined indications about the details of the surgical strategy. Route of access and the decision to clamp the renal hilum during enucleoresection depended on a variety of factors such as patient profile (general health status, comorbidities), tumor characteristics (size, location, complexity) and surgeon preference. In the early days of the robotic era in our hospital, smaller (cT1a), cortical and exophytic tumors that were located anteriorly, below or above the hilar plane were scheduled for robot-assisted NSS while more complicated tumors (hilar, cT1b-T2, mostly endophytic) were managed by open NSS in order to ensure a reasonable warm ischemia time. However, as our experience grew, we started doing robot-assisted NSS for more challenging tumors. This selection bias, which can be understandable within the context of the learning curve, should be considered while interpreting our results. Future prospective studies enrolling higher number of patients will more precisely highlight the importance of adapting preoperative morphometric evaluation into routine clinical practice.

## Conclusions

R.E.N.A.L. and P.A.D.U.A. systems influenced the way we handled localised renal masses. High R.E.N.A.L. and P.A.D.U.A. scores increased the likelihood of an open NSS. Although R.E.N.A.L. score differed significantly between surgical alternative subgroups, it did not exhibit sufficient statistical power to be a significant predictor of pedicle clamping during NSS. Morphometric evaluation, especially R.E.N.A.L. and P.A.D.U.A. systems, seem to have a clear impact on the decision-making process for the surgical treatment of localised renal tumors. Further prospective studies enrolling higher number of patients may establish the actual predictive power of morphometric scoring systems in the surgical planning of renal masses.

## Abbreviations

RCC: Renal cell carcinoma; NSS: Nephron-sparing surgery; RENAL: Radius, exophytic/endophytic, nearness, anterior/posterior, location; PADUA: Preoperative aspects and dimensions used for anatomic classification; C-index: Centrality index; ASA: American Society of Anesthesiologists; ONSS: Open nephron-sparing surgery; RANSS: Robot-assisted nephron-sparing surgery; EBL: Estimated blood loss; WIT: Warm-ischemia time.

## Competing interests

The authors declare that they have no conflict of interests.

## Authors’ contributions

TE: Conception and design, acquisition of data, analysis and interpretation of data, carried out morphometric scoring, critical revision of the manuscript for scientific and factual content, supervision. ÖA: Conception and design, acquisition of data, analysis and interpretation of data, statistical analysis, drafting the manuscript, critical revision of the manuscript for scientific and factual content. AM: Conception and design, acquisition of data, helped to draft the manuscript. MV: Conception and design, acquisition of data, analysis and interpretation of data, carried out morphometric scoring. All authors read and approved the final manuscript.

## Pre-publication history

The pre-publication history for this paper can be accessed here:

http://www.biomedcentral.com/1471-2490/13/63/prepub
